# Detection of Pediatric Upper Extremity Motor Activity and Deficits With Accelerometry

**DOI:** 10.1001/jamanetworkopen.2019.2970

**Published:** 2019-04-26

**Authors:** Catherine R. Hoyt, Andrew N. Van, Mario Ortega, Jonathan M. Koller, Elyse A. Everett, Annie L. Nguyen, Catherine E. Lang, Bradley L. Schlaggar, Nico U. F. Dosenbach

**Affiliations:** 1 Department of Neurology, Washington University School of Medicine in St Louis, St Louis, Missouri; 2 Program in Occupational Therapy, Washington University School of Medicine in St Louis, St Louis, Missouri; 3 Department of Psychiatry, Washington University School of Medicine in St Louis, St Louis, Missouri; 4 Program in Physical Therapy, Washington University School of Medicine in St Louis, St Louis, Missouri; 5 Department of Anatomy and Neurobiology, Washington University School of Medicine in St Louis, St Louis, Missouri; 6 Kennedy Krieger Institute, Baltimore, Maryland; 7 Department of Radiology, Washington University School of Medicine in St Louis, St Louis, Missouri; 8 Department of Pediatrics, Washington University School of Medicine in St Louis, St Louis, Missouri; 9 Department of Biomedical Engineering, Washington University School of Medicine in St Louis, St Louis, Missouri

## Abstract

**Importance:**

Affordable, quantitative methods to screen children for developmental delays are needed. Motor milestones can be an indicator of developmental delay and may be used to track developmental progress. Accelerometry offers a way to gather real-world information about pediatric motor behavior.

**Objective:**

To develop a referent cohort of pediatric accelerometry from bilateral upper extremities (UEs) and determine whether movement can accurately distinguish those with and without motor deficits.

**Design, Setting, and Participants:**

Children aged 0 to 17 years participated in a prospective cohort from December 8, 2014, to December 29, 2017. Children were recruited from Ranken Jordan Pediatric Bridge Hospital, Maryland Heights, Missouri, and Washington University School of Medicine in St Louis, St Louis, Missouri. Typically developing children were included as a referent cohort if they had no history of motor or neurological deficit; consecutive sampling and matching ensured equal representation of sex and age. Children with diagnosed asymmetric motor deficits were included in the motor impaired cohort.

**Exposures:**

Bilateral UE motor activity was measured using wrist-worn accelerometers for a total of 100 hours in 25-hour increments.

**Main Outcomes and Measures:**

To characterize bilateral UE motor activity in a referent cohort for the purpose of detecting irregularities in the future, total activity and the use ratio between UEs were used to describe typically developing children. Asymmetric impairment was classified using the mono-arm use index (MAUI) and bilateral-arm use index (BAUI) to quantify the acceleration of unilateral movements.

**Results:**

A total of 216 children enrolled, and 185 children were included in analysis. Of these, 156 were typically developing, with mean (SD) age 9.1 (5.1) years and 81 boys (52.0%). There were 29 children in the motor impaired cohort, with mean (SD) age 7.4 (4.4) years and 16 boys (55.2%). The combined MAUI and BAUI (mean [SD], 0.86 [0.005] and use ratio (mean [SD], 0.90 [0.008]) had similar F1 values. The area under the curve was also similar between the combined MAUI and BAUI (mean [SD], 0.98 [0.004]) and the use ratio (mean [SD], 0.98 [0.004]).

**Conclusions and Relevance:**

Bilateral UE movement as measured with accelerometry may provide a meaningful metric of real-world motor behavior across childhood. Screening in early childhood remains a challenge; MAUI may provide an effective method for clinicians to measure and visualize real-world motor behavior in children at risk for asymmetrical deficits.

## Key Points

**Question:**

Can accelerometry be used to measure typical development and identify motor deficits in childhood?

**Findings:**

In this cohort study of 185 children aged 0 to 17 years, age was a significant predictor of total activity as measured by bilateral upper extremity accelerometry. The mono-arm use index, which provides clinically relevant visualization of asymmetric impairment, is described.

**Meaning:**

Bilateral upper extremity accelerometry is an affordable, efficient method to objectively measure real-world movement to identify motor aberrancies in childhood.

## Teaser

This cohort study of pediatric patients uses accelerometry data from the upper extremities to evaluate whether movement can distinguish between children with typical development and those with motor deficits.

## Introduction

Developmental delays affect approximately 1 in 6 children in the United States and are a common medical issue seen by pediatric primary care professionals.^1^ The costs associated with disability are substantial and continue throughout life.^2,3^ To improve long-term outcomes, standard care has incorporated early developmental screening of infants and young children, leading to increased identification of delays and subsequent referral to appropriate services.^4,5^ Developmental delays in the first years of life can be subtle, difficult to detect by parents, and not immediately obvious in brief clinical encounters.^6,7,8,9,10,11,12^ Improving methods for the early detection of deficits would allow for earlier intervention during critical periods of rapid development and thus could reduce disability and associated costs.^13^

Motor development is the earliest observable benchmark of developmental progress because of its rapid, predictable advancement in young children. Developmental milestones in other domains are not as easily tracked and are difficult to measure at young ages.^14^ Arguably, then, motor development is the best target for early identification of more widespread disability. Yet accurate measurements of real-world motor behavior have been challenging. Hence, simple, affordable, and quantitative measurements of movement using wearable biosensors, such as accelerometers, during childhood could improve pediatric screenings for developmental delays.

Wearable technology is quickly becoming part of everyday life and has opened the possibility to objectively measure real-world behavior outside of the clinical environment.^15^ Wearable biosensors that measure acceleration allow for easy collection of large amounts of data about an individual’s activity.^16,17,18^ Accelerometry research in adults suggests it is reliable and valid^16,19,20,21,22^ with potential clinical relevance.^23^ Capturing real-world activity with accelerometers could be especially valuable for pediatric patients, as they often behave differently in the clinical setting.^24,25^ The potential for accelerometry in pediatrics has been recognized but has largely been limited to tracking physical activity and sleep disturbances,^26,27,28,29,30,31,32,33^ often relying on short wearing periods and hip-worn sensors in small patient populations.^34,35^ Accelerometers have not yet been used to detail typical motor development, to our knowledge.

Apart from accurately measuring general activity levels, we hypothesize that bilaterally worn accelerometers can also detect asymmetries in motor patterns. Deficits affecting one side of the body, or hemiparesis, constitute the most common form of cerebral palsy (CP), which is the most common cause of pediatric disability.^36,37^ Therefore, the early identification of real-life motor asymmetries could greatly facilitate diagnosis and treatment for this population. Conversely, children with identified brain injury (eg, perinatal stroke) are presumed to need rehabilitation services, although some have no neurological deficits. To date, there is limited ability to measure the real-world upper extremity (UE) activity with high interrater reliability.^38^ Previous methods that have analyzed UE movement have calculated the ratio of total frequency of movement of each UE, which can provide valuable information in adults or typically developing children.

In children, wrist-worn accelerometers encourage greater adherence and provide more accurate information about physical activity than hip-worn sensors.^39^ Collecting data during the course of several complete days on bilateral wrists from a referent cohort would allow for comparison between populations and the identification of children with aberrant motor patterns. Expanding the use of accelerometers to routine clinical care in populations at risk for motor and other developmental delays would provide greater understanding of children’s daily activity, allowing primary care professionals to address concerns quickly and with targeted interventions.

The purpose of this study was 2-fold: first, to gather and analyze bilateral UE accelerometry data from a referent cohort of children with typical motor development, and second, to test the validity of the referent bilateral UE accelerometry to discriminate between children with and without motor deficits. Since CP is the most common cause of motor disability in childhood and asymmetric deficits are the most common subtype of CP, we hypothesized that examining bilateral UE data and comparing them with unilateral UE movements would facilitate identification and diagnosis.

Our referent pediatric accelerometry (PEAC) data set represents more than 14 000 hours (561 days) from 156 children aged 0 to 17 years and provides a critical foundation for future studies to describe activity across childhood. Children with hemiparesis have asymmetric deficits that are obscured by traditional analysis and visualization methods, so we propose a new metric to separate unilateral and bilateral movement and incorporate acceleration of movement to more accurately describe the association of asymmetric deficits with real-world UE behavior, which may help classify children who would most benefit from intensive interventions. Isolating unilateral movements using the mono-arm use index (MAUI) should be able to objectively classify indicators for motor disability that are otherwise missed.

## Methods

The Human Research Protection Office of Washington University School of Medicine in St Louis approved this study. A prospective, observational cohort design was used to measure bilateral UE activity in 2 groups of children aged 0 to 17.11 years; children who were developing typically (referent cohort) and children with a diagnosis of asymmetric motor impairment (CP cohort) were recruited from December 8, 2014, to December 29, 2017. Parents provided written informed consent and children older than 7 years provided written assent. Children were asked to wear bilateral UE accelerometers for four 25-hour periods within 1 month. Child behavior, medical history, and demographic data were collected via parent report and managed using Research Electronic Data Capture tools.^40^ This study is reported following the Strengthening the Reporting of Observational Studies in Epidemiology (STROBE) reporting guideline.

### Participants and Procedures

Pediatric accelerometry participants were recruited using snowball sampling,^41^ in which consented individuals referred research members to other potential participants. Consecutive sampling and matching were used to ensure equal representation of age and sex, with a minimum of 8 children per age year. Children were included if they were typically developing with no significant medical history affecting motor development. Children with structural brain disease, neurological impairment, or autism spectrum disorder were excluded.

To confirm typical development, parents completed either the Movement Assessment Battery for Children-2 Checklist^42^ for children 5 years or older or the motor subscales of the Ages and Stages Questionnaire^43^ for children younger than 5 years. In addition to motor screening, the Child Behavior Checklist^44^ was completed for children older than 18 months. Scores from these measures were compared with published age norms. Parents of typically developing children older than 5 years completed the Participation and Environment Measure for Children-Youth^45,46^ to capture typical daily activities.

Participants with confirmed asymmetric motor deficits associated with CP were recruited through the pediatric neurology department at Washington University School of Medicine in St Louis, St Louis, Missouri, and Ranken Jordan Pediatric Bridge Hospital, Maryland Heights, Missouri. Children were excluded if they had autism spectrum disorder, received botulinum toxin therapy in the previous 3 months, or had undergone an orthopedic surgical procedure in the previous 6 months. We aimed to recruit at least 20 participants with hemiplegic CP classified by a gross motor function classification scale^47^ score of 1 or 2.

A pediatric occupational therapist assessed UE function of children to confirm asymmetric deficits using standardized assessment tools (eMethods in the Supplement). The Child Behavior Checklist^44^ was completed for children older than 18 months.

### Measurement of Bilateral Upper Extremity Activity

The ActiGraph wGT3X (ActiGraph LLC) is an accelerometer commonly used in pediatric research^48^ and was selected for this study because of its durability, long battery life, and water resistance. Children wore accelerometers bilaterally, just above the ulnar styloid, for four 25-hour periods within 1 month, with movement sampled at 30 Hz. The first and last 30 minutes of data were removed from each 25-hour period to allow for children getting used to the devices or taking them off a few minutes early. Children with at least 72 hours of recorded data were included in analysis (eMethods in the Supplement). By plotting the variance over the number of samples collected, we determined that 4 days was sufficient for stability of the activity count measurements (eFigure 1 in the Supplement). A single vector magnitude was calculated for each second by combining activity counts across axes (activity counts = √*x*^2^ + *y*^2^ + *z*^2^) and the resulting value was stored as activity counts (1 count = 0.001664*g*) in 1-second epochs and used for subsequent processing.^49^ Data were visually inspected in 30-minute increments for irregularities in activity counts or wear time to identify potential errors in data collection. Parents reported hand dominance of the child at the time of recruitment or close to the child’s third birthday.

### Data Processing and Analysis

Accelerometry data were processed using MATLAB version 2015a (The MathWorks Inc) and Python version 3.6 (Python Software Foundation). Periods of movement were determined using previously described methods.^22^

#### Referent Cohort 

Total activity was calculated by summing the seconds in which the activity count was greater than 10 for each UE. The sum from the dominant UE was used to calculate the total hours of activity for each 24-hour period. Methods for describing UE use and characterizing asymmetric deficits have traditionally relied on a ratio of the sum of seconds of movement of both UE, called the *use ratio* (UR). To characterize the contribution of each UE on a second-by-second basis, we calculated the UR, magnitude ratio, and the bilateral magnitude (eMethods in the Supplement).

#### Cerebral Palsy Cohort

The UR classifies each second as either movement or nonmovement. Using this parameter, bilateral contributions gain equal representation in the dominant/nondominant parts of the ratio, driving the value of the UR toward 1. Thus, the UR is a representation of unilateral movement and may be less sensitive to more subtle deficits because of its inclusion of bilateral data, which tend to be more frequent. Because we aimed to identify asymmetrical use of the UEs, a new, more sensitive metric was developed. The MAUI and bilateral-arm use index (BAUI) include the acceleration of movement such that the intensity of the movement is also taken into account, rather than the mere presence of activity. We also split the unilateral and bilateral contributions into separate indices to provide a more representative evaluation of the data.

#### Equations

Mono-arm use index and BAUI can be expressed with the following equations:
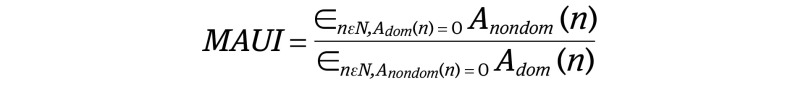


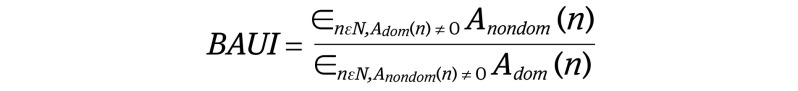
The sample, n, represents a single sample in the total number of samples, N. The activity count, A, represents the activity of the individual at a particular n and is separated between the dominant and nondominant extremities. By summing the activity counts across each extremity through each conditional sum, the use index, R, for the bilateral and unilateral contributions is obtained.

**Figure 1.**
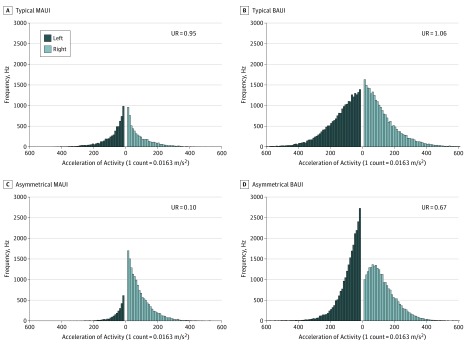
Activity From Upper Limbs in a Typically Developing Child and a Child with Asymmetric Deficits Histograms representing activity from both upper limbs in bins of 10 during 24 hours and representing two 8-year-old girls. A and B, Typically developing child who uses both limbs equally. C and D, A child with cerebral palsy. Her right hand is used more for independent movement (C), and her left upper limb is used predominantly in low-intensity bilateral movement (D). BAUI indicates bilateral-arm use index; MAUI, mono-arm use index; UR, use ratio.

The MAUI reflects the ratio of the sum of the magnitude of all independent movements of each arm. The BAUI reflects the ratio of the total intensity of bilateral UE movements. The MAUI more accurately describes the extent of deficit by objectively measuring the effort of each arm and quantifying the frequency of independent movement in everyday activities (eg, texting, opening doors). The UR obscures potentially informative data and is often visualized with complicated 3-dimensional plots. As illustrated in Figure 1, the MAUI and BAUI metrics provide an intuitive solution to visualize an individual’s movement that is missed using the UR.

### Statistical Analysis

Analyses were completed using R version 3.5.3 (R Project for Statistical Computing). The 2 cohorts were compared by age (*t* test), sex (χ^2^ test), and handedness (Fisher exact test). Total activity was summed for each day of accelerometry data and the UR between UE was calculated. A general additive model was used to curve fit the data and summary statistics were calculated. For the referent cohort, the mean and SD were calculated to summarize the UR and UE activity for each age year. The median magnitude ratio and bilateral magnitude were calculated for each age year. The magnitude ratio was calculated for each second of data by taking the natural log of the vector magnitude of the nondominant UE and dividing it by the vector magnitude of the dominant UE; a magnitude ratio of 0 reflects equal contribution from both UEs.^21,50^ The bilateral magnitude denotes the intensity of activity on a second-by-second basis by summing the vector magnitude of both the dominant and nondominant UEs; a bilateral magnitude of 0 reflects no activity.^21,50^ To describe differences in UE use, unpaired *t* tests were used to compare children older than 36 months (presumed handedness) with children younger than 36 months. To compare between the PEAC referent cohort and children with motor deficits, the MAUI and BAUI indices were calculated. We compared the performance of our proposed MAUI and BAUI metrics with the UE UR using logistic regression classification. To validate our model, we used stratified nested k × l-fold cross validation (k = 7, l = 7) for 30 trials, which was selected for computational tractability. For each trial, we recorded the mean F1 scores (measure of accuracy relying on precision and recall) and the area under the curve across the 7 k-folds. Then, the SD was calculated across trials to determine the effectiveness of using MAUI to accurately discriminate between those with and without asymmetric motor deficits.

## Results

### Participant Characteristics

Two cohorts were recruited for this study. A total of 216 children enrolled and 185 children were included in the analyses. The data for the PEAC cohort and associated code are publicly available online.^51^ The Table presents demographic information for both cohorts. Groups differed in in age (*t* = −2.77; *P* *=* .006) and handedness (*P* < .001 by Fisher exact test), but did not differ with respect to sex (χ^2^_1_ = 2.37; *P* = .12).

**Table.** Characteristics of Participants

Characteristic	No. (%)	
	Typically Developing (n = 156)	Motor Impaired (n = 29)
**Children**		
Boys	81 (52)	16 (55)
Age, mean (SD), mo	109 (61.61)	89 (52.7)
Right hand dominance	147 (94)	7 (24)
Race/ethnicity^a^		
White	141 (90)	27 (93)
Multiracial	9 (6)	0
African American	4 (3)	0
Asian	2 (1)	1 (3)
Hispanic or Latino	1 (1)	2 (7)
Not reported	0	1 (3)
Developmental score outside of clinical norms		
MABC^b^	2 (1)	NA
ASQ^b^	2 (1)	NA
CBCL^c^	4	5
Not reported	6 (4)	NA
**Parents**		
Marital status		
Married	137 (88)	25 (86)
Divorced or separated	11 (7)	2(7)
Single or not married	8 (5)	2 (7)
Maternal educational level		
Doctoral or professional degree	58 (37)	0
Bachelor’s or master’s degree	84 (54)	21 (72)
Associate degree or some college	13 (8)	6 (21)
High school diploma or GED equivalent	1 (0.1)	0
No. of children in home		
≥3	64 (41)	9 (31)
2	56 (36)	12 (41)
1	20 (13)	7 (24)
0 or not reported	16 (10)	1 (3)

Abbreviations: ASQ, Ages and Stages Questionnaire, fine and gross motor subtests for children age 0 to 5 years; CBCL, Child Behavior Checklist for children older than 5 years; GED, General Education Development; MABC, Movement Assessment Battery for Children Checklist for ages older than 5 years; NA, not applicable.

^a^ Race/ethnicity was self-reported. Participants were allowed to select Hispanic or Latino in addition to race/ethnicity, explaining why sum is more than 100%.

^b^ Parents completed surveys to confirm typical development.

^c^ Child Behavior Checklist for children older than 5 years; total scores greater than 2-fold SD were considered abnormal.

### Referent Cohort (PEAC)

Of the 176 typically developing children who were enrolled in the PEAC cohort, data from 2 participants were removed, 1 child for inaccurate device placement and 1 child because of observed developmental delay by one of us (C.R.H.). An additional 18 participants were removed because of insufficient data caused by suspected device malfunctioning, discrepancy between right and left recording length, or wear time. In the referent cohort, 156 children were included in analysis. The mean (SD) age was 9.1 (5.1) years, and there were 81 boys (52.0%). Overall, parents reported that their children participated predominantly in sedentary activities (eFigure 2 in the Supplement).

**Figure 2.**
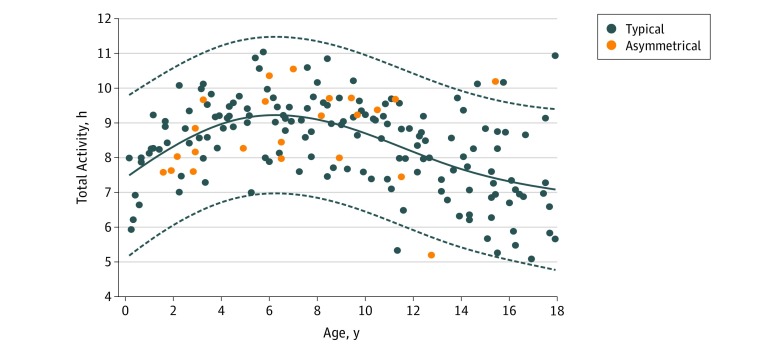
Upper Limb Movement of Measured With Bilateral Accelerometers Plot shows the association of age with total daily activity, measured in 1-second epochs. Each point represents a 24-hour period. The solid line represents a local regression fit of all visits using a general additive model, and dashed lines represent 95% CI.

Figure 2 illustrates the developmental curve using a general additive model with cubic smoothing splines of real-life daily activity, in which age was a significant predictor of total activity (*F* = 28.2, *R^2^* = 0.16, *P* < .001). Objective measurement of total activity across childhood in typically developing children is critical for beginning to understand changes in both active and sedentary behavior.

**Figure 3.**
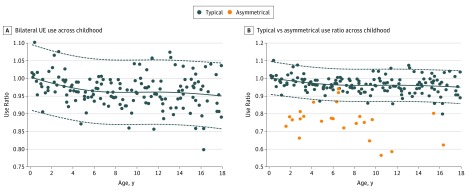
Use Ratio of Total Activity of Upper Limbs in Children Aged 0 to 17 Years Solid line represents a local regression of all pediatric accelerometry visits, and dashed lines represent 95% CI (referent cohort). A, Mean upper extremity use ratio declined in years of life. The decline demonstrated increased use of a dominant upper limb in typically developing children. B, Use ratio was lower for children with asymmetric impairment. Points within the 95% CI represent children without significant impairment (Video 2). UE indicates upper extremity.

The decline of the UE UR reported in Figure 3A represents the first objective measurement of bilateral UE use across childhood, to our knowledge. Children at the youngest end of the cohort had a mean UR of 1, which declined in the first years of life to reach adult norms by adolescence and can be observed in the MAUI ratio of typically developing children as they age (Video 1). The difference in UR between those with and without hand dominance (cutoff for handedness was set at 36 months) was statistically significant (*t* = −3.83, *P* < .001). These findings correspond with the age range when hand dominance is considered to emerge and solidify.^52^ Total activity hours and counts, UR, magnitude ratio, and bilateral magnitude are reported for each age year in the eTable in the Supplement.

**Video 1.** Upper Limb Activity of Typically Developing Children The histograms represent the activity distribution of unilateral movements of each upper limb for each visit in the typically developing cohort, presented from youngest to oldest.

### Cerebral Palsy Cohort

**Figure 4.**
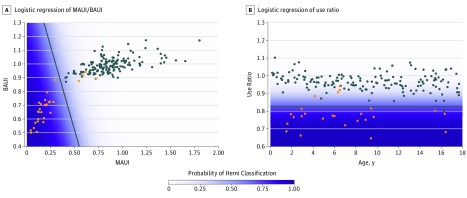
Unilateral and Bilateral Movements and Use Ratios Between Groups A, Using the mono-arm use index (MAUI) and bilateral-arm use index (BAUI) metric found an F1 score of 0.92 (SD, 0.008) and area under the curve of 0.98 (SD, 0.004). B, Using the use ratio methods from Bailey and Lang^22^ with the same data, there was an F1 score of 0.90 (SD, 0.008) and area under the curve of 0.98 (SD, 0.004).

Of the 40 children recruited for the CP cohort, 29 participants were included in the analysis. The mean (SD) age was 7.4 (4.4) years and 16 (55.2%) were boys. Participants were excluded if there were insufficient data (<72 hours). All motor deficits were associated with hemiplegic CP (gross motor function classification score of 1 or 2) due to various etiologies (eFigure 3 in the Supplement). Nine children (31%) were born prematurely, and 12 children (41%) had a documented learning disability. Children with asymmetric impairment demonstrated similar total activity to their typically developing peers (Figure 2) but used their dominant hands significantly more, which is observable in the differences in UR in Figure 3B. The combined MAUI and BAUI metric had a larger margin of separation when compared with the UR, with more differentiation carried by the MAUI metric (Figure 4). As seen in Figure 1, this separation can be easily visualized in individuals with asymmetric impairment. Using a logistic regression model (Figure 4) validated with our k × l-fold cross validation, we compared the effectiveness of the UR metric with the combined MAUI and BAUI metric. The combined MAUI and BAUI metric (mean [SD], 0.86 [0.005]) and UR (mean [SD], [0.008]) had comparable F1 values. The area under the curve was also comparable between the combined MAUI and BAUI metric (mean [SD], 0.98 [0.004]) and UR (mean [SD], 0.98 [0.004]). Video analysis from the Melbourne Assessment demonstrated that children misclassified by MAUI did not have measurable deficits impairing UE activity, as visualized with the child in Video 2, with Melbourne domain scores ranging from 85% to 100% (not significantly impaired). The MAUI demonstrated that children with asymmetric motor deficits used their dominant arms much more than their affected arms. We did not impose a minimum amount of total unilateral activity, which is the basis of the MAUI analysis. Therefore, since unilateral movement is a subset of total activity, variance across days was higher than for the total UR.

**Video 2.** Child With Cerebral Palsy (CP), Undetected With MAUI This video highlights a child who had CP yet did not demonstrate significant motor disability during clinical evaluation and were misclassified by our mono-arm use index (MAUI).

## Discussion

This study provides preliminary data that bilateral UE accelerometry can track typical childhood motor development and be used to discriminate those with subtle asymmetric motor impairments in CP. Given the importance of early diagnosis and the challenges associated with current screening methods, a reliable method for objectively measuring real-world motor behavior that is clinically useful is greatly needed. Our novel approach of measuring activity on bilateral UE in children using single-use bracelets had several important advantages. Primarily, we were able to acquire data continuously through all activities of daily living and eliminate participants having to remember to don devices following bathing or sleeping. Our findings provide promising evidence that wearable technology can provide medically important information across childhood, creating the possibility to screen at-risk populations (eg, premature infants) and determine the extent that brain injury may have affected real-world movement, helping to determine the need for intervention. Further, our measurement of bilateral activity in typically developing children allowed us to compare pediatric populations for the first time, to our knowledge, establishing that our cohort of children with asymmetric impairment had total activity that fell within normal limits compared with the PEAC cohort. Our comparison between UEs using the UR provided a clear indication that, while total activity have been similar, children with asymmetric impairment used their dominant hands more frequently and that our MAUI metric of the independent UE movements would provide a more accurate picture of UE motor disability.

Another important finding from this study is that accelerometry measured with wearable devices may provide a quantitative method to track developmental trajectories, such as the emergence of handedness, in addition to providing a useful clinical tool to describe real-world motor behavior and its association with child development. While it is possible to observe strong hand preferences at early ages, subtle differences in UE use are often difficult to identify. The low cost and low participant burden of wearable technology present an exciting opportunity to measure real-world motor behavior in the clinical setting.^15^ Using bilateral UE accelerometers greatly improved measurement of childhood motor activity by increasing the quality of our data (comparing UEs) and simultaneously providing the ability to track the UR across ages.

### Limitations

The present study was an observational cohort investigation of children with inherent limitations. To recruit this large cohort and meet family constraints, we were often reliant on parents to properly affix the accelerometers on their children. Although we are confident in the presented results, it is possible that accelerometers were used incorrectly. To meet recruitment goals, children were categorized by their age year, which limits analyses of discrete changes, especially in early childhood when development is rapid. Although we were cautious about not overfitting the data, it is possible that the reported efficacy of this model is optimistic. As is required for many developmental curves, a larger cohort of children in the first 2 years of life should be considered for future studies. Future studies should bolster the younger cohorts to get a better idea of when hand dominance emerges, if it can be reliably identified, and if disability can be predicted with accelerometry. Accelerometry data collected from children who participate in sports would further benefit future studies describing typical activity in older children.

## Conclusions

This is the first study to use accelerometers to measure activity from birth to adulthood and separate unilateral and bilateral movement, to our knowledge. The UR provides important information about UE activity. However, our findings indicate that separating the unilateral and bilateral movements using MAUI and BAUI metrics may be a more efficient method to screen for subtle motor aberrancies in childhood. However, we found that MAUI drives most cases and provides clinically relevant information that can be readily visualized and interpreted by the health care team to identify children who present with atypical motor patterns. Our findings indicate that our MAUI metric may be a useful tool in pediatric neurologic and rehabilitative care even at very young ages. The misclassified measurements were from children with impairments diagnosed from magnetic resonance imaging findings and who had been referred for intensive unilateral rehabilitation. However, despite neural damage, these children presented motor skills similar to typical peers (Video 2), indicating that a costly, time-consuming intervention may not be warranted.

## Notes

Supplement. **eMethods. **Motor Assessment of Children with Deficits **eReferences.** **eTable.** Referent Accelerometry Data of Typically Developing Cohort **eFigure 1.** Variance of Activity Over Time **eFigure 2.** Most Common Daily Activities of Typically Developing Children Older Than 5 Years **eFigure 3.** Causes Associated With Motor Deficits in Cohort
