# Supplementary material for: Detection of Pediatric Upper Extremity Motor Activity and Deficits With Accelerometry
Source: JAMA Netw Open. 2019 Apr 26;2(4):e192970. doi: 10.1001/jamanetworkopen.2019.2970 (PMC6487720; doi:10.1001/jamanetworkopen.2019.2970)
Supplement: Supplement. — eMethods. Motor Assessment of Children with Deficits eReferences. eTable. Referent Accelerometry Data of Typically Developing Cohort eFigure 1. Variance of Activity Over Time eFigure 2. Most Common Daily Activities of Typically Developing Children Older Than 5 Years eFigure 3. Causes Associated With Motor Deficits in Cohort [file jamanetwopen-2-e192970-s001.pdf]

Supplementary Online Content

Hoyt CR, Van AN, Ortega M, et al. Detection of pediatric upper extremity motor activity and deficits with accelerometry. *JAMA Netw Open*. 2019;2(4):e192970. doi:10.1001/jamanetworkopen.2019.2970

eMethods. Motor Assessment of Children with Deficits
eReferences.
eTable 1. Referent Accelerometry Data of Typically Developing Cohort
eFigure 1. Variance of Activity Over Time
eFigure 2. Most Common Daily Activities of Typically Developing Children Older Than 5 Years
eFigure 3. Causes Associated With Motor Deficits in Cohort

This supplementary material has been provided by the authors to give readers additional information about their work.

© 2019 Hoyt CR et al. *JAMA Network Open.*

eMethods. Motor Assessment of Children with Deficits

A pediatric occupational therapist assessed upper limb function of children with CP using the Melbourne Test of Unilateral Upper Limb Function-2[1](MA). The MA is reliable and validated assessment of the quality of upper limb movement in children >2.5 years with neuromotor impairment. The MA consists of 14 items that were videotaped and independently scored by two trained graduate students (M.B., S.G.) blinded to the child's diagnosis. Movement is scored based on four domains: accuracy, range of motion, dexterity and fluency resulting in a percent score for each category. Children <2.5 years completed the Quality of Upper Extremity Skills Test (QUEST).[2] The QUEST consists of 36 items that are scored and summarized in four categories: dissociated movements, grasp, protective extension and weight bearing.

Accelerometry Protocol

Compliance

In addition to wrist placement[3], we took several additional steps to maximize wearing compliance. In contrast to previous pediatric studies, the accelerometers were worn for the full 25-hour period, including sleep and bathing, to reduce errors in placement and to more fully describe daily activity. Parents selected wearing periods and children could select colorful paper or plastic wristbands with optional stickers to decorate the devices. A printed visual instruction sheet was provided with extra wristbands to maximize protocol adherence. Color-coding and labels were used to facilitate placement on the correct upper limb. Lastly, parents were offered a reminder text or email to ensure adherence to scheduled wearing time. For later reference, a link to an instructional video was emailed to the parent. Devices were returned directly to team members or mailed using a prepaid envelope. Gift cards were provided for each 25-hour period.

Data Quality

To ensure data quality, we carefully examined each recording period. The first and last 30 minutes of data were removed from each 25-hour sample to allow for a period of adjustment or early removal. In the typically developing cohort, wearing periods were removed if total activity counts were <10,000 or >20 million because of suspected device malfunction.[4] Further, wear time was calculated using the ActiLife software and data were removed if wear time was less than 90% of the recording period or if there was >3% discrepancy between upper limb (UL) wear time.[5]

Accelerometry Variables

© 2019 Hoyt CR et al. *JAMA Network Open.*

Accelerometry data from typically developing children were characterized by the use ratio, magnitude ratio and bilateral magnitude. The use ratio is calculated by dividing the activity counts of the non-dominant limb by the activity counts of the dominant limb. Therefore, a use ratio of 1 indicates equal use, and <1 would indicate more use of the dominant limb which is reflected in reported norms of 0.95 ± 0.06 in healthy adults.[6] The magnitude ratio was calculated for each second of data by taking the natural log of the vector magnitude of the non-dominant upper limb and dividing it by the vector magnitude of the dominant upper limb. The bilateral magnitude signifies the intensity of acceleration on a second by second basis by summing the vector magnitude of both the dominant and non-dominant upper limb; higher bilateral magnitude values indicate increasing intensity of upper limb activity.[6,7] For example, higher values with larger, faster movements (e.g. shooting a basketball) whereas lower values reflect less intense movement (e.g. playing on a hand-held device).

eReferences

1. Randall M, Carlin JB, Chondros P, Reddihough D. Reliability of the Melbourne assessment of unilateral upper limb function. *Developmental medicine and child neurology.* 2001;43(11):761-767.
2. DeMatteo C, Law M, Russell D, Pollock N, Rosenbaum P, Walter S. The reliability and validity of Quality of Upper Extremity Skills Test. *Physical and Occupational Therapy in Pediatrics.* 1993;13(2):1-18.
3. Fairclough SJ, Noonan R, Rowlands AV, Van Hees V, Knowles Z, Boddy LM. Wear Compliance and Activity in Children Wearing Wrist- and Hip-Mounted Accelerometers. *Medicine and science in sports and exercise.* 2016;48(2):245-253.
4. Crawford D, Cleland V, Timperio A, et al. The longitudinal influence of home and neighbourhood environments on children's body mass index and physical activity over 5 years: the CLAN study. *Int J Obesity.* 2010;34(7):1177-1187.
5. Choi L, Liu Z, Matthews CE, Buchowski MS. Validation of accelerometer wear and nonwear time classification algorithm. *Medicine and science in sports and exercise.* 2011;43(2):357-364.
6. Bailey RR, Klaesner JW, Lang CE. An accelerometry-based methodology for assessment of real-world bilateral upper extremity activity. *PloS one.* 2014;9(7):e103135.
7. Bailey RR, Klaesner JW, Lang CE. Quantifying real-world upper-limb activity in nondisabled adults and adults with chronic stroke. *Neurorehabilitation and neural repair.* 2015;29(10):969-978.

© 2019 Hoyt CR et al. *JAMA Network Open.*

eTable 1. Referent Accelerometry Data of Typically Developing Cohort

Age (yr.)	(visit)	Hours Activity (24 hrs.) mean (SD)	Activity Counts ND mean (SD)	Activity Counts D mean (SD)	Use Ratio ND/D mean (SD)	Bilateral Magnitude IQR (25th, 75th)	Magnitude Ratio IQR (25th, 75th)
0	8(30)	8.64 (1.36)	25,633 (4,464)	25,320 (4,707)	1.02(0.05)	(144, 169)	(-0.006, 0.071)
1	8(31)	10.06 (0.78)	29,967 (2,615)	30,756 (2,721)	0.98 (0.04)	(177, 205)	(-0.062, -0.006)
2	7(27)	9.90 (1.46)	29,914 (5,431)	29,540 (5,619)	1.02 (0.05)	(196, 218)	(-0.001, 0.042)
3	11(42)	10.46 (1.50)	31,689 (4,830)	32,570 (5,049)	0.97 (0.04)	(214, 236)	(-0.047, 0.017)
4	7(25)	10.78 (0.79)	31,814 (2,650)	33,422 (2,752)	0.95 (0.04)	(201, 225)	(-0.075, 0.026)
5	9(34)	10.96 (1.50)	32,740 (5,462)	34,359 (5,273)	0.95 (0.04)	(219, 246)	(-0.042, 0.018)
6	10(38)	10.58 (1.06)	31,466 (3,646)	32,313 (3,794)	0.98 (0.05)	(210, 237)	(-0.047, 0.065)
7	8(31)	10.48 (1.09)	31,203 (3,835)	32,327 (3,909)	0.96 (0.04)	(205, 243)	(-0.033, 0.024)
8	8(27)	10.58 (1.24)	31,314 (4,430)	32,925 (4,629)	0.95 (0.05)	(223, 248)	(-0.043, 0.024)
9	9(33)	10.44 (1.33)	30,889 (5,012)	32,406 (4,194)	0.95 (0.06)	(210, 250)	(-0.053, 0.018)
10	9(35)	10.50 (1.12)	30,726 (3,673)	31,375 (4,139)	0.98 (0.06)	(218, 240)	(-0.025, 0.046)
11	9(34)	9.62 (1.92)	27,841 (6,927)	28,727 (6,342)	0.96 (0.07)	(210, 244)	(-0.059, 0.079)
12	8(28)	9.95 (1.23)	28,027 (3,912)	29,809 (4,360)	0.94 (0.06)	(202, 224)	(-0.072, 0.020)
13	9(32)	9.15 (1.96)	26,917 (6,765)	26,610 (6,769)	0.98 (0.08)	(201, 221)	(-0.034, 0.084)
14	9(32)	9.72 (1.80)	27,828 (5,861)	28,704 (6,065)	0.97 (0.06)	(200, 228)	(-0.064, 0.039)
15	11(44)	9.26 (2.15)	26,016 (7,129)	27,127 (7,372)	0.96 (0.07)	(198, 223)	(-0.043, 0.078)
16	8(45)	8.22 (1.50)	22,407 (5,090)	23,766 (5,127)	0.95 (0.09)	(193, 240)	(-0.079, 0.056)
17	8(25)	9.10 (2.31)	25,392	26,553	0.95 (0.07)	(184, 215)	(-0.016, 0.055)

© 2019 Hoyt CR et al. *JAMA Network Open.*

	(8,462)	(7,434)

© 2019 Hoyt CR et al. *JAMA Network Open.*

eFigure 1. Variance of Activity Over Time

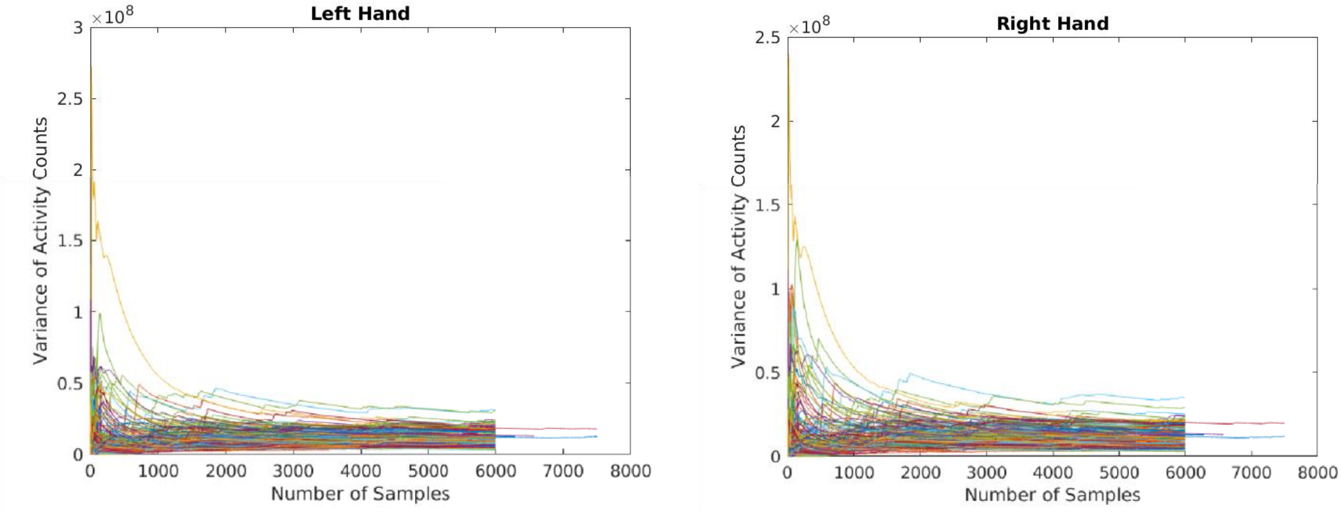

These figures represent the variance of activity over multiple days of sampling from bilateral upper limbs. Using accelerometry data summarized in activity counts and summed for each minute, a plateau in the variance can be observed after approximately 48 hours, or 2,880 samples, of data collection.

© 2019 Hoyt CR et al. *JAMA Network Open.*

eFigure 2. Most Common Daily Activities of Typically Developing Children Older Than 5 Years

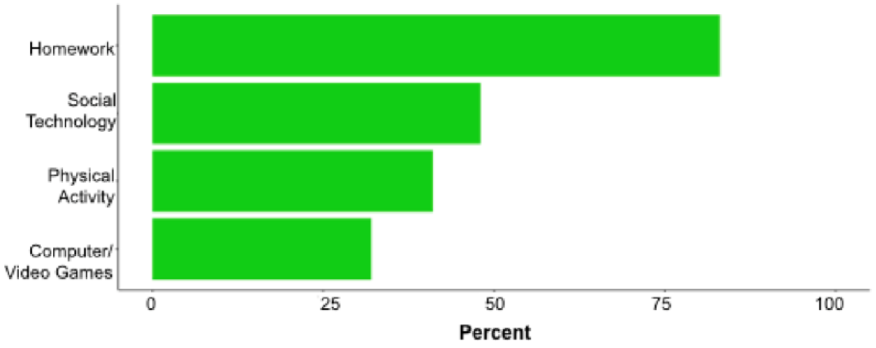

Parents reported activities that their child engages in daily in the PEM-CY. Typically developing children engaged predominantly in sedentary activities daily.

eFigure 3. Causes Associated with Motor Deficits in Cohort 2

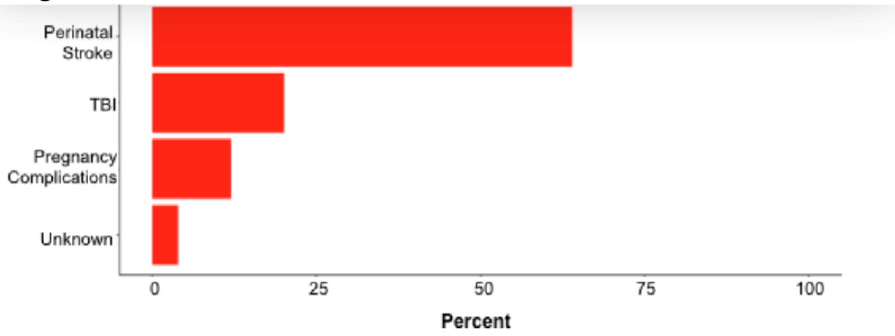

Parents reported suspected causes of motor deficits in medical history survey. The majority were associated with complications related to perinatal stroke.

© 2019 Hoyt CR et al. *JAMA Network Open.*
